# Impact of ‘decision-to-delivery’ interval on maternal and perinatal outcomes: a retrospective study of emergency caesarean section from 2017 to 2021 at a secondary health facility in Nigeria

**DOI:** 10.1186/s12884-024-06700-y

**Published:** 2024-07-22

**Authors:** Mariam Abdulbaki, Fullaila O Aliyu, Musa Ayinde, Amudalat Issa, Abiodun S Adeniran, Olayinka R Ibrahim

**Affiliations:** 1 Department of Obstetrics and Gynecology, General Hospital Ilorin, Ilorin, Kwara State Nigeria; 2 Department of Clinical Pharmacy and Pharmacy Practice, Faculty of Pharmaceutical Science, University of Ilorin, Ilorin, Kwara State Nigeria; 3 Children Specialist Hospital Ilorin, Ilorin, Kwara State Nigeria; 4 Department of Obstetrics and Gynecology, University of Ilorin Teaching Hospital, Ilorin, Kwara State Nigeria; 5 Department of Pediatrics, Division of Clinical Medicine, University of Global Health Equity, Kigali, Rwanda

**Keywords:** Emergency caesarean section, Decision-to-delivery interval, Maternal outcomes, Perinatal outcomes, Resource constraint settings

## Abstract

**Background:**

The decision-to-delivery interval (DDI) for a caesarean section is among the factors that reflect the quality of care a pregnant woman receives and the impact on maternal and foetal outcomes and should not exceed 30 min especially for Category 1 National Institute for Health and Care Excellence (NICE) guidelines. Herein, we evaluated the effect of decision-to-delivery interval on the maternal and perinatal outcomes among emergency caesarean deliveries at a secondary health facility in north-central Nigeria.

**Methods:**

We conducted a four-year retrospective descriptive analysis of all emergency caesarean sections at a secondary health facility in north-central Nigeria. We included pregnant mothers who had emergency caesarean delivery at the study site from February 10, 2017, to February 9, 2021.

**Results:**

Out of 582 who underwent an emergency caesarean section, 550 (94.5%) had a delayed decision-to-delivery interval. The factors associated with delayed decision-to-delivery interval included educational levels (both parents), maternal occupation, and booking status. The delayed decision-to-delivery interval was associated with an increase in perinatal deaths with an odds ratio (OR) of 6.9 (95% CI, 3.166 to 15.040), and increased odds of Special Care Baby Unit (SCBU) admissions (OR 9.8, 95% CI 2.417 to 39.333). Among the maternal outcomes, delayed decision-to-delivery interval was associated with increased odds of sepsis (OR 4.2, 95% CI 1.960 to 8.933), hypotension (OR 3.8, 95% 1.626 TO 9.035), and cardiac arrest (OR 19.5, 95% CI 4.634 to 82.059).

**Conclusion:**

This study shows a very low optimum DDI, which was associated with educational levels, maternal occupation, and booking status. The delayed DDI increased the odds of perinatal deaths, SCBU admission, and maternal-related complications.

## Introduction

Caesarean section (CS) is one of the oldest and most performed major surgical operations in modern Obstetrics [1]. It is a life-saving obstetric surgical procedure carried out on a pregnant woman after the age of viability, either electively or when there is an immediate threat to the life of the foetus and or the pregnant woman with the sole aim of delivering the foetus(es) and the placenta [2]. Though there is a great aversion to caesarean section in developing countries like Nigeria, it remains a life-saving procedure that has played a significant role in reducing foetal, maternal, and perinatal morbidity and mortality [3].

Amongst factors that affect the maternal and perinatal outcomes for an emergency caesarean section is the period between the decision taken and the actual time a baby is delivered, otherwise referred to as the decision-to-delivery interval (DDI) [3]. The DDI is also among the factors that reflect the quality of care a pregnant woman receives, and it should not exceed 30 min [4, 5]. When the DDI exceeds 30 min, it constitutes a third-degree delay and is associated with an increased risk of maternal and perinatal morbidity and mortality [6]. Studies have shown that achieving a normal decision-to-delivery interval has been very difficult, especially in resource-constraint settings, resulting in maternal and foetal complications, including deaths while awaiting caesarean Sects. [6, 7]. Although the historic standard was to achieve a DDI of 30 min for an emergency caesarean section to prevent adverse maternal and foetal outcomes, the recently updated National Institute for Health and Care Excellence (NICE) guidelines recommend that the condition of the pregnant woman and her unborn child be taken into account when making a decision about an emergency caesarean section, as rapid birth can be harmful in certain circumstances [8]. The Royal College of Obstetricians and Gynaecologists (RCOG) and the recently updated NICE guideline further subclassified the DDI for caesarean sections based on the degree of urgency due to the need for consideration of other factors that affect maternal and perinatal outcomes into four Categories [8]; Category 1: An immediate threat to the mother’s or the foetus’s life; potential uterine rupture; significant placental abruption; cord prolapse; prolonged foetal bradycardia; foetal hypoxia; Category 2: Foetal or maternal compromise that does not pose an urgent threat to life; Category 3: When there is no compromise for the mother or the foetus but an early birth is required. Category 4: Timed birth to suit the mother or medical professional. Though there are a few studies in Nigeria that evaluate the DDI and its impact on maternal and perinatal outcomes, these are mostly limited to tertiary health facilities, which may not be reflective of events in secondary health facilities [6, 7, 9]. This is because despite being closer to the people, the available resources, including personnel at most secondary health facilities in Nigeria, are far less when compared with tertiary health facilities, which may impact on the outcomes. Therefore, we determined the DDI and its impact on maternal and perinatal outcomes in a secondary health facility in north-central Nigeria. The outcomes of the study area will contribute to the planning and formulation of health policies and protocols that will reduce adverse maternal and perinatal outcomes.

## Methods

### Study area and settings

This study was carried out at the General Hospital Ilorin, a secondary health facility located centrally in the city of Ilorin, north-central Nigeria. Ilorin is the capital city of Kwara State, one of the six states in the north-central geopolitical zone of Nigeria, with a population estimate of 1,030,000 in 2023 [10]. The general hospital Ilorin offers specialist care and serves as a referral centre to the people of Kwara State and parts of the neighbouring states of Kogi, Niger, and Oyo. The hospital has an obstetric and gynaecological complex consisting of a 20-bed antenatal, 20-bed post-natal medical, and 20-bed gynaecological wards. This health facility has 24-hour coverage of the labour ward by medical officers, residents, and consultants. The obstetrics and gynecological complex is fully equipped with ultrasound, a fetal assessment unit, a delivery suit, an obstetric theatre, a special care baby unit, a pharmacy, and functional laboratories.

### Study design and population

This was a retrospective descriptive analysis of all the emergency caesarean sections carried out at the General Hospital Ilorin between February 10, 2017, and February 9, 2021. Records from the special care baby unit were also analysed as part of the perinatal outcomes. All parturient who received antenatal care in our facility were considered as “booked,” while those who received antenatal care in other healthcare facilities or did not receive antenatal care were considered ‘unbooked’. All labour cases were managed actively using a partograph, and the consultant made the final decision for the caesarean section. All caesarean sections were performed by at least a registrar or a senior medical officer.

### Sample size estimation

Using a proportion of 5.7% [11] among emergency caesarean section who did not have delayed DDI in a Nigerian study, we obtained a minimum sample size of 326 at a power of 95% and 2.5% level of precision using an online sample size calculator (http://www.raosoft.com/samplesize.html) [12].

### Inclusion and exclusion criteria

We included pregnant women who had a caesarean section at 28 weeks gestational age and beyond, irrespective of the outcomes. Women who had an elective caesarean section, assisted delivery, or vaginal delivery or those with gestational age less than 28 weeks were excluded from this study.

### Data collection

Data were extracted from the facility’s hard copy of delivery registers which contained the following information: name, age, address, phone number, folder number, booking status, mode of delivery (spontaneous vaginal delivery, instrumental delivery, or Caesarean section), presentation (emergency or elective caesarean section), gravidity, parity, number of babies alive, number of stillbirths, number of abortion, time of delivery, sex of baby, APGAR score, and birth weight, whether baby was transferred to the special care baby units or nursed by the mother’s bedside. The period of data collection extended from twenty-eight weeks of gestational age up to six weeks postpartum.

### Instruments and data collection procedure

The principal investigator and four trained research assistants reviewed all delivery records and folders from February 2017 to February 2021, and extracted information from those who had emergency caesarean sections. The parameters reviewed for this study included the age of the parturient, estimated gestational age, gravidity, parity, previous history of caesarean section (CS), indications for caesarean section, the decision-to-delivery interval, maternal and perinatal morbidity, and mortality. Data was collected using structured proforma, which was serialised to avoid multiple entries. Where there are two or more indications for caesarean section, a single indication was assigned according to the international classification of diseases (clinical modification, ninth edition, ICD-9-CM) hierarchical categories.

### Data analysis

Data were entered into a personal computer, which was pass-worded and analysed using SPSS version 22. The age was summarised as mean with standard deviations and further sub-grouped for analysis. The other socio-demographic variables were summarized as percentage and frequency. The decision-to-delivery interval (DDI) was expressed as the proportion of women who had emergency CS within 30 min and expressed as a percentage. The association between the factors that contributed to delayed DDI was evaluated using Chi-square and Fischer’s exact tests as appropriate. The impact of the DDI on maternal and perinatal outcomes was evaluated with an odds ratio along with a 95% confidence interval, and all levels of statistical significance were set at a value less than 0.05.

### Ethical consideration

Ethical approval was obtained from the ethical review committee of the General Hospital Ilorin Health Research Committee (GHI/IRC/246/VOL.I/99). The ethical review committee waived the informed consent for this study because it was a retrospective study, and the data were anonymized. All the retrieved data were de-identified.

## Results

This study involved 582 pregnant women who had emergency CS during the study period. The overall mean age was 29.2 (5.8) years, and most were between ages 26 to 30 years (40.7%) and had a tertiary level of education (59.8%). The commonest gestational age group among the study participants was term (37 to 42 completed weeks; 69.2%), and most were booked (88.0%). Also, 98.6% had direct family payments for the CS, and the mechanism for payment for a caesarean section is activated at the point of diagnosis (Table 1). The major indications for emergency CS were cephalopelvic disproportion (157;27.0%), failure to progress (91; 15.6%), and foetal distress (66;11.3%), as shown in Table 2.

**Table 1:** Socio-demographic characteristics of parturient that had a caesarean section

Variable	Frequency *n* = 582	Percent (%)
**Age group (Years)**		
≤ 25	149	25.6
26–30	237	40.7
31–35	101	17.4
36–40	79	13.6
> 40	16	2.7
**Mean ± SD (years)**	29.22 ± 5.76	
**Educational level**		
No formal education	8	1.4
Primary	28	4.8
Secondary	198	34.0
Tertiary	348	59.8
**Occupation**		
Artisan	48	8.2
Civil servant	104	17.9
Skilled work	148	25.4
Trading	161	27.7
Unemployed	97	16.7
Others	24	4.1
**Level education husband/partner**		
No formal education	12	2.1
Primary	20	3.4
Secondary	142	24.4
Tertiary	408	70.1
**EGA (weeks)**		
<37	175	30.1
37–42	403	69.2
>42	4	0.7
**Booking Status**		
Booked	512	88.0
Un-booked	70	12.0
**Gravidity**		
Primigravida	222	38.1
2–4	280	48.1
>4	80	13.8
Mode of payment*		
Family payment	574	98.6
Health insurance	8	1.4

*The mechanism for payment for caesarean section is activated at the point of diagnosis

**Table 2:** Indication for Cesarean Section

Variable	Frequency *n* = 582	Percentage
**Indications** (multiple response)		
Cord Prolapse + Live Baby	12	2.1
Cephalopelvic disproportion in labour	157	27.0
Failure to Progress	91	15.6
Fetal distress	66	11.3
Severe preeclampsia	64	11.0
Bleeding Placenta Praevia	44	7.6
>1 previous scar in labour	31	5.3
Breech in labour	24	4.1
Obstructed labor	64	11.0
Others	37	6.4

### Decision-to-delivery interval

Out of the 582 pregnant women who underwent CS in this study, only 32 (5.5%) had CS within less than or equal to 30 min. All parturient 32 (5.5%), who had normal DDI, paid all their hospital bills from their ‘family pocket’ while all 8 (1.4%) who had health insurance coverage experienced delayed DDI (Table 1). This study’s median DDI time was 200.00 min (Interquartile ranges from 140.00 to 320.00 min), with a minimum time of 15.00 min and a maximum of 1925.00 min.

### Maternal, foetal, and neonatal outcomes

The most common poor maternal outcomes in this study were anaemia (148; 25.4%), postpartum haemorrhage (96; 16.5%), sepsis (81; 13.9%), and wound infection (81; 13.9%) and among the anesthetic-related complications included hypotension (52; 8.9%) and post-spinal headache (29; 5.0%), as shown in Table 3.

**Table 3:** Maternal and perinatal outcomes (multiple response) of the emergency CS

Variable	Frequency *n* = 582	Percentage (%)
**Maternal outcomes**		
Postpartum hemorrhage	96	16.5
Sepsis	81	13.9
Anemia	148	25.4
Wound infection	81	13.9
**Anesthetic related complications**		
Hypotension	52	8.9
Post-spinal headache	29	5.0
Cardiac arrest	8	1.4
**Fetal and neonatal outcomes**		
SCBU admission*	176	30.2
Days of admissions**		
-Less or equal 7 days	132	75.0
-8 days or more	44	25.0
Overall deaths (foetal and neonatal)*	56	9.6
Deaths from SCBU admission**	12	6.8

SCBU-special care baby unit; *Total Emergency CS as denominator (582);** total admissions into SCBU as denominator (176)

Based on perinatal outcomes, of the 582 emergency CS, 56 foetal deaths occurred (9.6%), and 176 (30.2%) babies had special care baby unit (SCBU) admission. Among the babies admitted, the median admission duration was 2 days (IQR 2 to 7.8), and there were 12 deaths (6.8%) of the 176 admissions (Table 3).

### Factors associated with delayed DDI

The factors that were significantly associated with delayed DDI included educational levels (both the pregnant women and their spouses), maternal occupation, and booking status (Table 4).

**Table 4:** Factors that are associated with delayed DDI in the study participants

Variable	No Delayed DDI *n* = 32	Delayed DDI *n* = 550	c^2^	*P* value
**Age (years)**				
≤ 25	8 (25.0)	141 (25.6)	1.257f	0.861
26–30	16 (50.0)	221 (40.2)		
31–35	4 (12.5)	75 (13.6)		
36–40	0 (0.0)	16 (2.9)		
**Educ. level**				
No formal educ.	0 (0.0)	8 (1.5)	22.149f	< 0.001
Primary	0 (0.0)	28 (5.1)		
Secondary	24 (75.0)	174 (31.6)		
Tertiary	8 (25.0)	340 (61.8)		
**Occupation**				
Artisan	0 (0.0))	48 (8.7)	56.660f	< 0.001
Civil servant	4 (12.5)	100 (18.2)		
Skilled work	0 (0.0)	148 (26.9)		
Trading	12 (37.5)	149 (27.1)		
Unemployed	4 (12.5)	93 (16.9)		
Others	12 (37.5)	12 (2.2)		
**Level educ. husband/partner**				
No formal educ.	4 (12.5)	8 (1.5)	24.013	< 0.001
Primary	0 (0.0)	20 (3.6)		
Secondary	16 (50.0)	126 (22.9)		
Tertiary	12 (37.5)	396 (72.0)		
**EGA (weeks)**				
<37	8 (25.0)	167 (30.4)	0.572f	0.655
37–42	24 (75.0)	379 (68.9)		
>42	0 (0.0)	4 (0.7)		
**Booking Status**				
Booked	20 (62.5)	492 (89.5)	20.765	< 0.001
Un-booked	12 (37.5)	58 (10.5)		
**Gravidity**				
Primigravida	32 (100.0)	490 (89.1)	4.103f	0.135
2–4	0 (0.0)	56 (10.2)		
>4	0 (0.0)	4 (0.7)		
Mode of payment				
Family payment	32 (100.0)	542 (98.5)	0.472f	1.000
Health insurance	0 (0.0)	8 (1.5)		

Educ-educational; f-Fischer exact tests. EGA-Estimated gestational age

### Association between delayed DDI, maternal and perinatal outcomes

The delayed DDI was associated increase in perinatal death (foetal and neonatal) with an odds ratio (OR) of 6.9 (95% CI, 3.166 to 15.040) and increased odds of SCBU admissions (OR 9.8, 95% CI 2.417 to 39.333) as shown in Table 5. Among the maternal outcomes, delayed DDI was associated with increased odds of sepsis (OR 4.2, 95% CI 1.960 to 8.933), anesthetic-related complications of hypotension (OR 3.8, 95% 1.626 TO 9.035), and cardiac arrest (OR 19.5, 95% CI 4.634 to 82.059).

**Table 5:** Association between Delay DDI and poor maternal, fetal and neonatal outcomes

Variable				COR	95% CI	*P*
DDI	Perinatal outcomes					
		**Not Admitted**	**Admitted**			
**≤ 30 min**	**Total ECS delivery**	20 (4.9%)	(12 (6.8%)	1		
**> 30 min**		386 (95.1%)	164 (93.2%)	1.412	0.675, 2.956	0.428
		**Discharged**	Deaths**			
**≤ 30 min**	**Total ECS delivery**	20 (3.8%)	12 (21.4%)	1	3.166, 15.040	
**> 30 min**		506 (96.2%)	44 (78.6%)	6.900		< 0.001
		**Discharged**	**Deaths**			
**≤ 30 min**	**SCBU admissions**	8 (4.9%)	4 (33.3%)	1	2.417, 39.333	
**> 30 min**		156 (95.1%)	8 (66.7%)	9.750		0.005
	**Maternal outcomes**					
		**Yes**	**No**			
**≤ 30 min**	**Postpartum hemorrhage**	8 (8.3)	24 (4.9)	1		
**> 30 min**		88 (91.7)	462 (95.1)	1.750	0.762, 4.021	0.217
**≤ 30 min**	**Sepsis**	12 (14.8)	20 (4.0)	1		
**> 30 min**		69 (85.2)	481 (96.0)	4.183	1.960, 8.933	< 0.001
**≤ 30 min**	**Anaemia**	8 (5.4)	24 (5.5)	1		
**> 30 min**		140 (94.6)	410 (94.5)	0.976	0.429, 2.222	1.000
**≤ 30 min**	**Wound infections**	8 (9.9)	24 (4.8)	1		
**> 30 min**		73 (90.1)	477 (95.2)	2.178	0.943. 5.031	0.069
**≤ 30 min**	Hypotension***	8 (15.4)	24 (4.5)	1		
**> 30 min**		44 (84.6)	506 (95.5)	3.833	1.626, 9.035	0.005
**≤ 30 min**	Cardiac arrest***	4 (50.0)	28 (4.9)	1	4.634, 82.059	
**> 30 min**		4 (50.0)	546 (95.1)	19.500		< 0.001

DDI-Decision delivery interval; COR-Crude odds ratio; CI-Confidence interval; ECS-Emergency caesarean section; SCBU-Special care baby unit; **total deaths (foetal and neonatal); #Chi square results; ***anesthetic related complications

## Discussion

This study shows a very low rate of optimum DDI based on the American College of Obstetricians and Gynaecologists (ACOG) and the Royal College of Obstetricians and Gynaecologists (RCOG) recommendation of 30 min [13]. Worthy of note is the fact that all of the parturient in this study group belong to Category I NICE Categorization of CS urgency, in which case there is an immediate threat to the life of the mother or the foetus(es) [8]; hence, a DDI of 30 min is most suitable to achieve optimal maternal and perinatal outcomes. The median decision-to-delivery interval in this study was also longer than the recommended time. Out of the 582 pregnant women who underwent CS in this study, only 32 (5.5%) had CS within 30 min of decision-to-delivery interval with a minimum time of 15.00 min. The observation is similar to studies carried out in some tertiary hospitals in Ilorin [6] and Ife [7], both in Nigeria, Kenya [14], and Uganda [15]. The similarities in our findings compared to the previous studies may be related because these studies were conducted in low-income countries with similar socioeconomic indices such as low socioeconomic status, levels of education of both the pregnant women and their spouses, maternal occupation, and booking status. This study also showed a significant association between delayed DDI and levels of education (parturient and their partners/spouses), occupation, and booking status. Studies have shown that a higher level of education improves the ability to comprehend and take decisions within a short period of time regarding delivery and caesarean Sects. [16, 17]. A local study also showed that achieving an optimal DDI is higher in booked parturients when compared to unbooked parturients, underscoring the role of adequate antenatal care in birth preparedness and complication awareness and readiness by providing adequate information and education on the benefits of early presentation in labour, anticipated labour complications, and treatment modalities [6]. A similar study carried out in Easter Uganda showed the effect of maternal occupation on the DDI with a higher percentage of parturients who are not gainfully employed having a longer DDI, underscoring the importance of maternal occupation on the DDI [15]. In addition, health system challenges such as high patient load, low staffing, and recurrent out-of-stock medical supplies are more prevalent in resource-constraining settings, including the study site, and may have partly contributed to delayed DDI for emergency CS observed in this study [18]. In Nigeria, enrolment in the National Health Insurance Scheme (NHIS) may not guarantee a normal DDI due to the challenges facing the NHIS, some of which include inadequate facilities and staffing, a few or non-availability of NHIS-accredited centres in rural areas, the non-availability of most prescribed drugs, delays and bottlenecks with enrolment, and a lack of accessibility to NHIS staff during call hours [19].

In contrast to our observations in this study, studies carried out in Ethiopia [20] and Cameroon [21] showed that 17.5% and 20.0%, respectively, of emergency caesarean deliveries achieved the proposed DDI of less than 30 min. The higher rate of emergency CS within 30 min compared to our study may reflect differences in healthcare systems across some African countries. However, a study carried out in South Africa [22] showed that no emergency caesarean delivery conformed to a DDI of less than 30 min, which was attributed to over diagnosis. The import of our finding is the urgent need to improve accessibility to the caesarean section with a focus on achieving a DDI of less than 30 based on the global recommendation not only at a tertiary health facility but also at the secondary facility. A DDI of more than 30 min in Category I NICE criteria constitutes a phase 3 delay in providing emergency obstetric care and has been linked to adverse maternal and foetal outcomes [8]. Hence, the commonest poor maternal outcomes observed in this study were anaemia, postpartum haemorrhage, wound infection and sepsis, with four times the odds of sepsis, about one time odd of anaemia, two times the odds of wound infection, 19.5 times the odds of cardiac arrest, and 1.7 times the odds of postpartum haemorrhage and this is comparable to studies done in Nigeria which shows adverse maternal outcomes with delayed DDI [6, 7]. The high prevalence of postpartum anaemia in this study may be due to the high burden of pre-delivery anaemia, primary postpartum haemorrhage, wound infections and post-op sepsis, which are more prevalent conditions among pregnant women in low- and middle-income countries [23]. This finding is similar to the report by Butwick et al. [24], which showed that postpartum haemorrhage and pre-delivery anaemia are strong independent risk factors for severe postpartum anaemia. These findings suggest the need to closely monitor women undergoing emergency CS for various complications, especially when DDI is delayed beyond the recommended time in resource-constraint settings like ours.

This study also showed that Delayed DDI was associated with adverse perinatal outcomes (increased SCBU admissions and foetal and neonatal deaths) with an almost 10 times odds of SCBU admission. Similar studies conducted in Nigeria [6.7], Somaliland [25], and Uganda [26], also showed increased perinatal deaths due to delayed DDI. The poor perinatal outcomes in this study are not unexpected because factors such as prolonged labour due to delayed DDI may lead to perinatal asphyxia and increased risk for neonatal sepsis, with a subsequent negative impact on the health of the foetus and newborn after birth. In contrast, studies carried out in Enugu [27] and Kano [28], both in Nigeria and Ethiopia [29], showed no differences in maternal and perinatal outcomes following delayed DDI. The differences between our findings compared with the studies in Enugu, Kano, and Ethiopia may be due to the fact our study was carried out in a typical secondary health facility with many challenges compared with the latter, which were tertiary health facilities, which may have fewer challenges. Some of the challenges observed at our facility included a few personnel, delayed surgery due to delayed payment as most of our patients paid out of pocket, and delayed consent due to society’s aversion to CS. The findings of poor perinatal outcomes observed in this study also called for sustained calls to achieve a global set of less than 30 min for DDI, identify barriers towards its implementation, and provide appropriate intervention at all levels of care in Nigeria.

### Limitations

This study has a few limitations. Firstly, this was a retrospective review of our data with a few missing incomplete records [22] that were excluded. Secondly, post-mortem was not done for the babies that died to ascertain the actual cause of death as their parents and relatives declined consent. While delayed DDI contributes to adverse maternal and foetal outcomes for emergency caesarean sections, it is also worthy of note that there are some other factors that may also influence maternal and perinatal outcomes that were not included in our study. Finally, this is a single-centre study at a secondary health facility, and the findings may not be generalized to the whole Ilorin metropolis.

## Conclusion

This study shows a very low optimum DDI, which was associated with educational levels (both the pregnant women and their spouses), maternal occupation, and booking status. The delayed DDI increased the odds of perinatal deaths, SCBU admission, and maternal-related complications. We recommend an urgent evaluation of factors contributing to delayed DDI, which can be addressed to improve the maternal and perinatal indices of emergency CS, especially at the secondary healthcare levels.

## Data Availability

The datasets used during this current study are available from the corresponding author on reasonable request.

## Abbreviations

DDI: Decision-to-delivery interval
CS: Caesarean Section
SCBU: Special Care Baby Unit
